# Monitoring abundance of aggregated animals (Florida manatees) using an unmanned aerial system (UAS)

**DOI:** 10.1038/s41598-021-92437-z

**Published:** 2021-06-21

**Authors:** Holly H. Edwards, Jeffrey A. Hostetler, Bradley M. Stith, Julien Martin

**Affiliations:** 1 Florida Fish and Wildlife Conservation Commission, Florida Fish and Wildlife Research Institute, Saint Petersburg, FL 33701 USA; 2 Independent Researcher, 7920 NW 71 St., Gainesville, FL 32653 USA; 3 U.S. Geological Survey, Wetland and Aquatic Research Center, Gainesville, FL 32653 USA; 4 U.S. Fish and Wildlife Service, 11510 American Holly Drive, Laurel, MD 20708 USA

**Keywords:** Ecology, Zoology

## Abstract

Imperfect detection is an important problem when counting wildlife, but new technologies such as unmanned aerial systems (UAS) can help overcome this obstacle. We used data collected by a UAS and a Bayesian closed capture-mark-recapture model to estimate abundance and distribution while accounting for imperfect detection of aggregated Florida manatees (*Trichechus manatus latirostris*) at thermal refuges to assess use of current and new warmwater sources in winter. Our UAS hovered for 10 min and recorded 4 K video over sites in Collier County, FL. Open-source software was used to create recapture histories for 10- and 6-min time periods. Mean estimates of probability of detection for 1-min intervals at each canal varied by survey and ranged between 0.05 and 0.92. Overall, detection probability for sites varied between 0.62 and 1.00 across surveys and length of video (6 and 10 min). Abundance varied by survey and location, and estimates indicated that distribution changed over time, with use of the novel source of warmwater increasing over time. The highest cumulative estimate occurred in the coldest winter, 2018 (N = 158, CI 141–190). Methods here reduced survey costs, increased safety and obtained rigorous abundance estimates at aggregation sites previously too difficult to monitor.

## Introduction

Quantifying groups of animals is important for modeling population dynamics and for management purposes; however, estimating the size of large groups remains challenging. An important problem with counting any animal species is imperfect detection of individuals, where animals are missed by observers because of perception bias or they cannot be observed because they are under vegetation cover or water (availability bias)^1^. Furthermore, some species gather in relatively large numbers in very tight spaces, which can prevent observers from detecting nearby conspecifics, and aquatic species often dive and resurface in a way that makes it difficult to tell individuals apart to get an accurate count. These errors can lead to underestimation of the number of individuals within a group because of imperfect detection. Over estimating is also possible but less common and occurs when the same individuals are misidentified or counted multiple times. Although numerous models have been developed to help estimate wildlife abundance while accounting for imperfect detection, large aggregations make the application of most existing protocols and models difficult or impossible to implement. Some of these models require scientists to be able to identify individual animals (e.g., spatial capture-mark-recapture), and others are affected by non-independence of detection or are just not appropriate to use when the data include multiple counts of the same animal.

Many taxa, including penguins, hippopotamus, pinnipeds and migrating terrestrial birds, form large aggregations (> 1000). These gatherings can be associated with a variety of behaviors such as foraging (e.g., seabirds, ungulates), breeding (e.g., penguins, pinnipeds), predator avoidance (e.g., roosting snail kites, passerines, sandhill cranes, and other species) and thermoregulation (e.g., bats, manatees)^2^. For many wildlife species, aggregating in large numbers can confer a fitness advantage, because it can reduce the risk of predation and vulnerability to other threats such as environmental disturbances or hunting and provide other benefits to individuals^2–6^.

Manatees also aggregate in large numbers to thermoregulate at power station discharge canals, natural springs, and passive thermal basins^7^. Manatees are temperature sensitive, when ambient water temperatures fall below about 18 °C, they can succumb to cold stress syndrome, which can lead to, for example, emaciation, myocardial degeneration, gastrointestinal tract infections, skin lesions or death^8^. To avoid cold stress, manatees overwinter at locations that provide water warmer than 20 °C^9^. The water at many of these aggregation sites is turbid, and since manatees can remain submerged for more than 15 min and groups at a single power plant can number in the thousands (Pers. Comm., K. Scolardi, Mote Marine Laboratory, written communication 1/24/2020; FWC unpublished data), accurately estimating their numbers can be challenging.

Some obstacles associated with estimating the size of large aggregations of animals can be overcome using unmanned aerial systems (UAS) or drone technology. Fixed-wing aircraft can be expensive, risky to operate or in some places, they can even be logistically difficult to procure, but UAS or drones are becoming easy and safe to use and provide a cost-efficient means to collect data on populations^10–12^ Although the use of UAS is increasing, airspace restrictions in many locations including the United States (https://www.faa.gov/uas/) have limited their scientific use to places that do not interfere with other air traffic or that are not considered “restricted.” Partly because of these limitations, the use of drones as a tool in ecology is still emerging, and most published papers have focused on the development and testing of applications more than the application itself. Much of the work involving UAS has focused on obtaining interesting photos or videos of target species, assessing the performance of equipment used for collecting data, the behavioral response of species to the overflight of the UAS (unmanned aerial vehicle) itself and on the ability of UAS to adequately detect the species of interest^13–17^. Few studies to date have been used to monitor species over large geographic areas or to implement multi-year studies to assess abundance and distribution of wildlife^11,12^.

In this study, we demonstrate how a UAS can be used to collect data to help avoid some of the logistical and detection issues associated with estimating the numbers of aggregated animals^18^. We show how to optimize the survey effort and to model UAS-collected data to estimate abundance and distribution while accounting for imperfect detection of a highly aggregated species, the Florida manatee (*Trichechus manatus latirostris*). We illustrate our innovative methodology by using it to monitor manatee abundance and distribution at several passive thermal refuges, naturally occurring bodies of water above 18 °C used by manatees to thermoregulate in winter^9^.

### Study area

**Figure 1:**
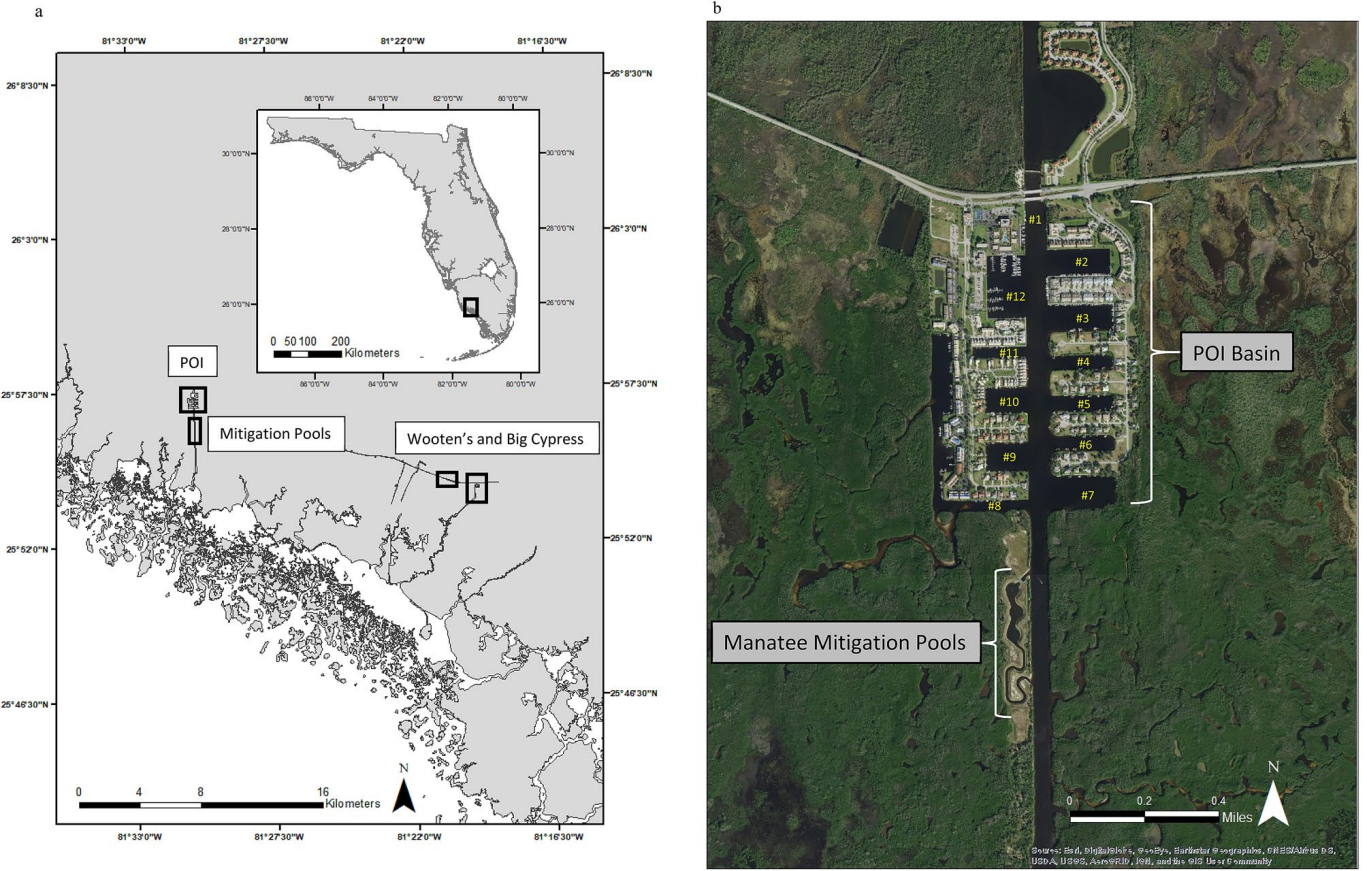
(**a**) Locator map of manatee aggregation sites (Port of the Islands (POI), Wooten’s Pond, Big Cypress National Preserve and the manatee mitigation pools) in the Ten Thousand Islands of south Florida, USA, (**b**) POI Basin and location of the 12 canals and mitigation pools surveyed by the UAS. Map generated in ArcMap 10.3 (https://www.esri.com).

Port of the Islands (POI) and nearby thermal sites (Wooten’s Pond and Big Cypress National Preserve) in the Ten Thousand Islands region of Collier County, Florida, are the overwintering locations of about 300 manatees (FWC unpublished data; Fig. 1a). The POI basin is the largest passive thermal basin used by Florida manatees for survival in winter (Fig. 1a,b). Freshwater input into the basin, drained from surrounding areas via a canal system, forms a halocline or sharp vertical salinity gradient that creates a barrier to convective mixing^19^. This halocline holds warmer saline bottom water (above 20 °C) below the colder freshwater surface lens, warm enough to sustain manatees in winter^19^. However, restoration of wetlands and changes in water flow to this basin as part of the Comprehensive Everglades Restoration Plan’s (CERP) Picayune Strand Restoration Project (PSRP) are expected to result in the loss of this warm-water habitat for manatees. To avoid adverse impacts to the population of manatees overwintering at this site and to provide sufficient warm-water habitat in the POI area, the U.S. Army Corps of Engineers and the South Florida Water Management District have created a manatee mitigation feature (Fig. 1a,b) consisting of an oxbow and three large pools, 6.1 m deep, with about 3 acres of water surface area (~ 12,141 m^2^) that taps into the brackish aquifer below it to provide warm water above 20 °C to the pools—warm enough for manatees to avoid cold stress. At the time of this research, the mitigation pools were open to manatees; however, water from the canals that helps create the original passive thermal basin had not been eliminated and the halocline was still intact. Therefore, water warmer than ambient temperature was still available to manatees in the basin.

To help ensure the best outcome for manatees and to inform future conservation efforts that may involve creating warmwater habitat for them, it is necessary to (1) optimally monitor and document the impacts of the restoration project (to be completed after 2023) on manatee distribution, and numbers (2) assess the viability of the implemented mitigation measures by determining if the mitigation pools provide adequate winter habitat that manatees are willing to use to avoid cold stress. This research addresses the first step of the project, to develop a means of effectively estimating manatee abundance and mapping distribution at the current local aggregation sites—POI boat basin (total), Big Cypress National Preserve, Wooten’s Pond (a small privately owned body of water northeast of POI) and the POI mitigation pools—before restoration is completed and the halocline is eliminated. To accomplish this, we developed an innovative closed mark-recapture method using a Bayesian hierarchical framework to estimate manatee abundance and detection from count data at each aggregation site using a UAS. This information will serve as a baseline for measuring distribution and abundance to help scientist and resource managers determine the impact of the restoration project on manatee use of warm-water sites in the POI region, including the new mitigation pools.

## Methods

### UAS surveys

**Figure 2:**
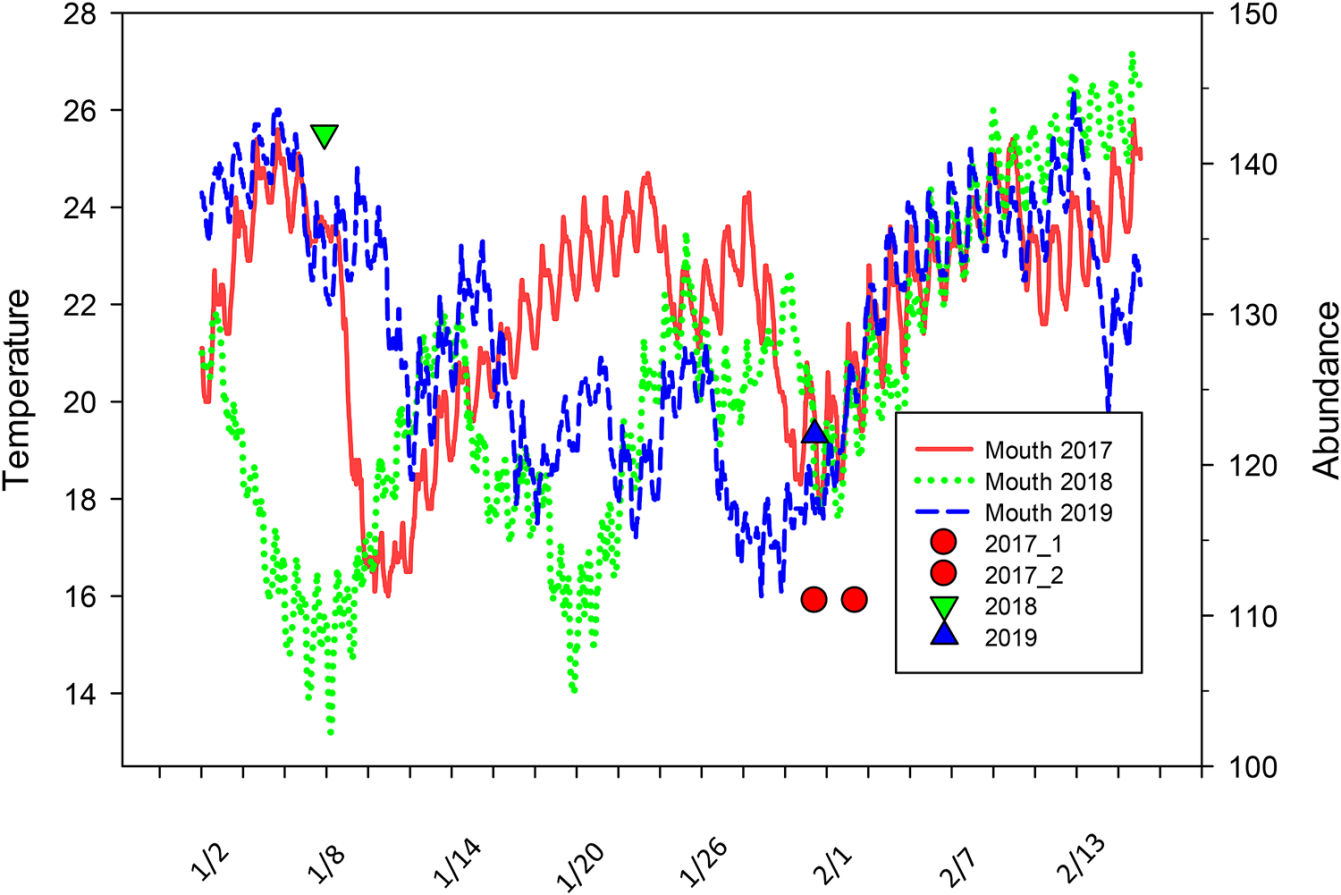
Water temperatures from the mouth of the Faka Union Canal south of the mitigation pools and estimates of manatee abundance during each survey in 2017, 2018 and 2019. Lines indicate water temperature: red line (2017), green dashed (2018) and blue dashed (2019). Shapes indicate the date of the survey and the abundance estimate: red circle (2017), green upside-down triangle (2018) and blue triangle (2019).

We conducted surveys from a standard DJI Phantom 4 Pro (P4P) UAS version 2 quadcopter during the winters of 2017 (31 Jan. and 2 Feb.), 2018 (7 Jan.), and 2019 (31 Jan.). The surveys were conducted following strong cold fronts when manatees were aggregated at the survey sites (Figs. 1a,b, 2). The use of the UAS platform allowed us to cover the area completely and safely, and the quality of the photos and video obtained was much superior to that from a fixed-winged aircraft. During a manatee fixed-wing survey, a Cessna 172 is usually flown at an altitude of about ~ 229 m and 70–90 kts. One or two observers are seated on the same side of the aircraft with the window open^20^. These platforms can be unstable (bouncy) and when tightly circling they can be difficult to keep over a target area, making it hard to take good photographs out an open window. Since the aircraft is moving, usually circling, target animals appear in different locations in each photograph making it difficult to distinguish individuals for analyses.

UAS surveys were conducted from about 8:30 a.m. to 4:30 p.m. eastern time. The UAS was launched and landed manually from ground locations near each canal. The UAS was flown manually to an altitude of 122 m, the maximum allowed by the FAA without special waivers, over each of the 12 POI canals, Wooten’s Pond, Big Cypress (2019 only) and the mitigation pools (Fig. 1a,b). The UAS was positioned manually over each canal to minimize glare and glint, and to ensure complete visual coverage, using the real time video downlink displayed on a hooded, 9″ tablet. The camera used was the built-in P4P camera, which has a 1″ (13.2 mm wide × 8.8 mm high) 20 megapixel CMOS sensor with a 24 mm wide angle lens (35 mm equivalent), giving a GSD of 3.34 cm/pixel when at nadir (pointed vertically) at 122 m altitude, and a camera footprint of approximately 183 m horizontally by 122 m vertically. Most canals could be fully captured at nadir, but several canals were taller than could be covered at nadir, requiring the drone to be repositioned and camera angle to be manually rotated off nadir until the entire canal, including reference points such as docks or seawalls were covered. Flying at 122 m allowed us to view all water area of each east–west canal numbered in Fig. 1b without moving the UAS, which hovered in a stationary position over each location for 10 min when manatees were present. If no manatees or mud plumes were seen by the UAS operator during the first 6 min, the flight was terminated. While hovering, the UAS collected continuous ultra-high definition (UHD) video, also known as 4 K video. As opposed to the fixed wing-aircraft, the video recorded from the UAS was stationary, stable and always over the desired target area, even in 20 mph gusty winds.

### Video processing

The video clips were captured at 4 K resolution with H.265 compression in MP4 files. The video files for each site were imported into a nonlinear video software package (DaVinci Resolve, Blackmagic Design Pty. Ltd.), merged as necessary to create a single file per site (files were sometimes split without time gaps on the UAS because of a 4 GB limit per file), passed through a dehazing filter (to improve contrast and saturation) and exported to new down scaled clips at HD (1080p) resolution for ease of handling. The clips were used to create a recapture history at 1-min intervals for capture-recapture analyses of individual manatees present at each of the locations.

**Figure 3:**
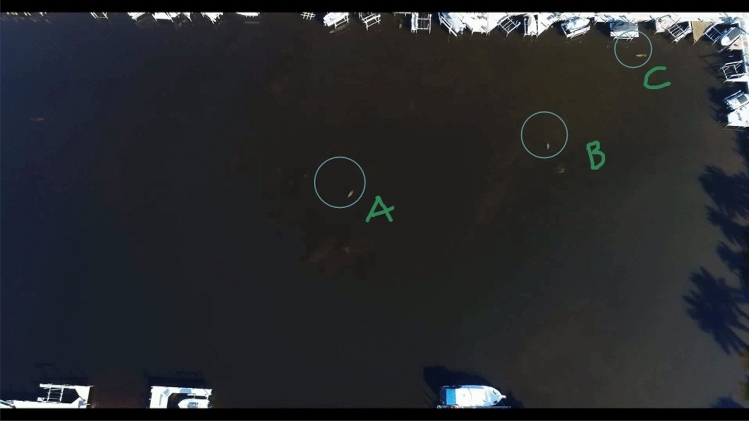
Example of manatees identified using the Kinovea software to create a sighting history for 1 min of video.

The video was reviewed by a single observer using open-source video analytical software, Kinovea, designed to help assess sports performance (www.kinovea.org). This software was used to identify and track manatees by annotating the video as each manatee became visible on the screen. For each of the 10 min (in 1-min intervals) of hover time, individual manatees were identified and labeled on the video with a letter designating their identity (Fig. 3). Because of the poor water clarity, height of the drone, and because manatees show only part of their torso during surfacing intervals, we were not able to identify them by individual markings. To help us identify animals on screen by their position in the basin and track any movement, we taped a plastic overhead projector transparency sheet on to the computer screen and used a “Sharpie” marker to mark the direction of the head and body position of each animal as it become visible on the video. The transparency was marked with the same letter that was used to annotate an individual on the video. The transparency was left in place for the entire video, which helped us identify new individuals by comparing their orientation and location to other individuals previously detected. Resting manatees normally surface briefly before sinking in the same location to the bottom to rest or they rest stationary on the surface for long periods of time^21^. Although some movement occurred during the time of the video, their movement could be tracked using the method of marking their location on a transparency on the screen.

A recapture history was created for each location separately by identifying which individuals (by position as described above) were available each min of film for 10 min. We also created a capture history for 6 min (1-min intervals) of the video for comparative purposes to assess if using a shorter time window (6 min versus 10 min) would provide acceptable results. The longer the period of observation, the more likely manatees are to move. Using fewer min in the capture history may decrease the possibility of making an identification error, would shorten operation time and battery life and it would help to optimize lighting conditions by maintaining the sampling window at times with a higher sun angle. The resulting capture history data were used to estimate the number of manatees in each canal.

### Analyses

We used a Bayesian closed capture-mark-recapture (CMR) model to estimate the number of manatees in each location and visit while accounting for imperfect detection^22,23^. We estimated average detection probability by visit and allowed detection to vary by location *l* and time *t* (in min) using a beta-binomial capture-mark-recapture model^24–26^ to account for possible non-independent detection from, for example, correlated manatee surfacing behavior:

1 $$\begin{aligned} & y_{{v,l,t,i}} \sim \text{Bern} \left( {z_{{v,l,i}} \cdot p_{{v,l,t}} } \right) \\ & p_{{v,l,t}} \sim \text{beta} \left( {\alpha _{v} ,\beta _{v} } \right), \\ \end{aligned}$$

where *y*_*v,l,I,t*_ was 1 if manatee *i* at location *l* and visit *v* was seen in min *t* and 0 otherwise; *z*_*v,l,i*_ was 1 if such a manatee existed (see below); *p*_*v,l,t*_ was the detection probability for visit *v*, location *l*, and min *t*; and *α*_*v*_ and *β*_*v*_ were the beta parameters that determine the mean ($\bar{p}_{v} )$ and correlation of detection probability $\left( {\rho _{v} } \right)$ in visit *v*^25,27,28^:

2 $$\begin{aligned} & \bar{p}_{v} = \frac{{\alpha _{v} }}{{\alpha _{v} + \beta _{v} }} \\ & \rho _{v} = \frac{1}{{\alpha _{v} + \beta _{v} + 1}}. \\ \end{aligned}$$

We used data augmentation to estimate abundance^23,29,30^ where we augmented the capture histories described in Video Processing with all-zero rows so that all site/visit combinations had a total of 100 rows and then modeled *z*_*v,l,i*_ for each row as Bernoulli distributed, with inclusion probability Ω:

3 $$z_{{v,l,i}} \sim Bern\left( \Omega \right).$$

The estimated abundance for location *l* and visit *v* is the sum of all such *z*:

4 $$N_{{v,l}} = \sum\limits_{{i = 1}}^{{100}} {z_{{v,l,i}} } .$$

Further derived parameters include *M*_*v*_, the total abundance for a visit across locations (we excluded Big Cypress from the totals because it was surveyed only in the 2019 visit).

We fit this model separately to the 6-min and 10-min capture histories in program JAGS^31^ using the R (R Development Core Team 2017^32^) package jagsUI^31^ as an interface. We ran three Markov Chain Monte Carlo (MCMC) chains for 120,000 iterations after a burn-in of 60,000 iterations and saw no signs of lack of convergence from Gelman-Rubin statistics or visual examination of the chains. We present the means of the posterior probability distributions as point estimates and the 2.5% and 97.5% quantiles as credible intervals.

## Results

### Estimates of detection

#### Detection probability for 1-min intervals

**Figure 4:**
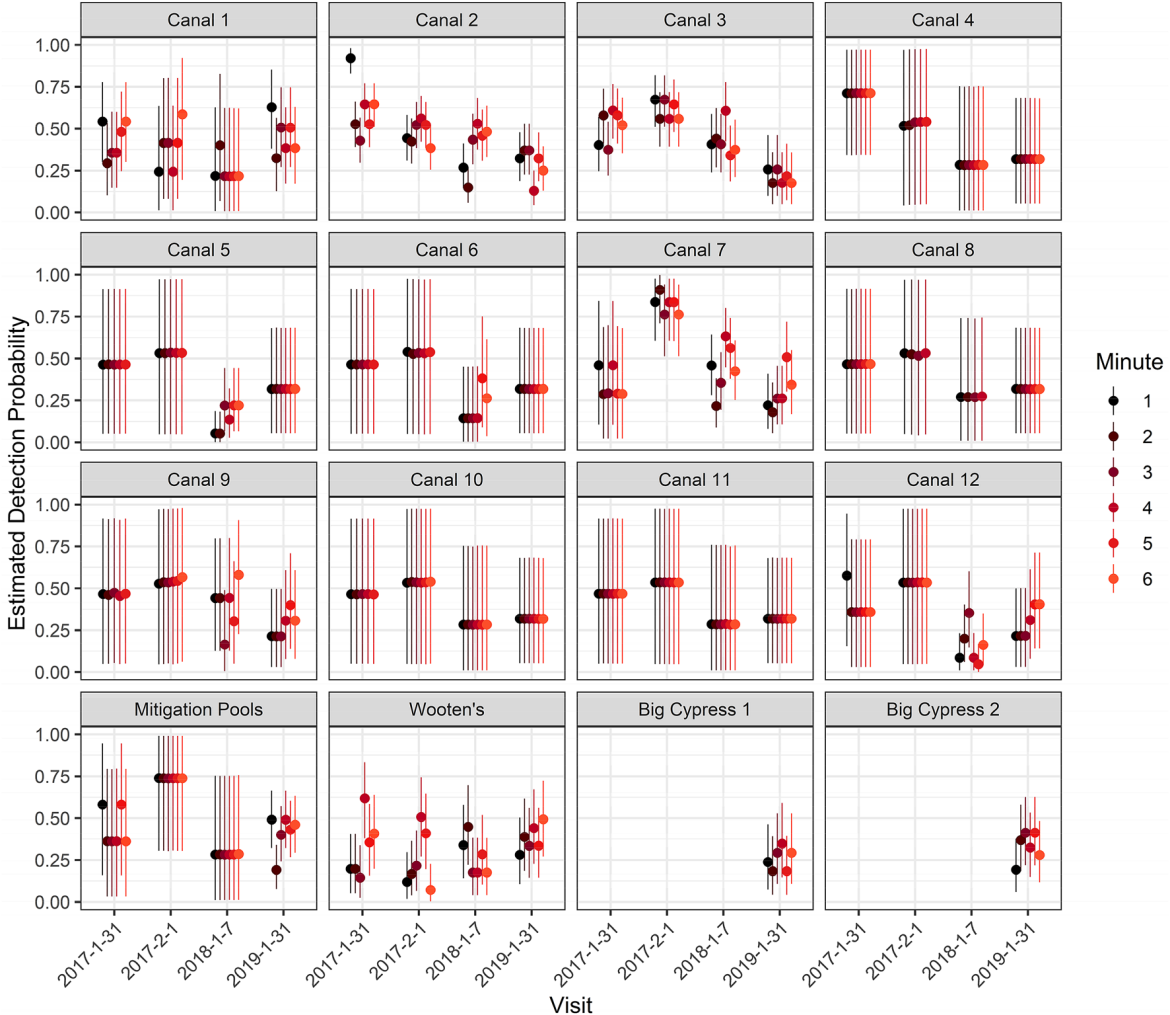
Estimates of the probability of detection for 1-min intervals for 6 min by site, date and min. Dots indicate means (point estimates) and lines the 95% credible intervals.

**Figure 5:**
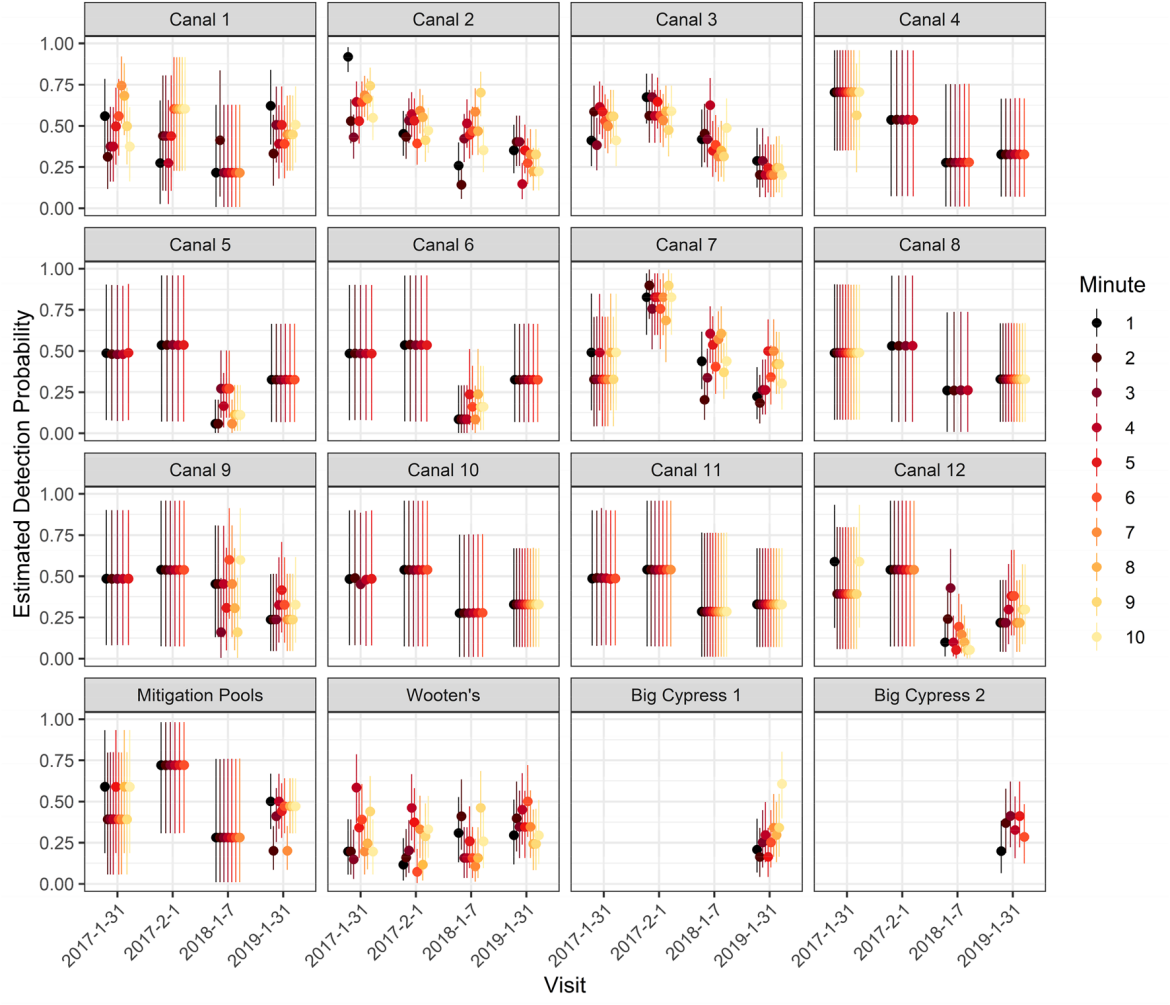
Estimates of the probability of detection for 1-min intervals for 10 min by site, date, and min. Dots indicate means (point estimates) and lines the 95% credible intervals.

Estimates of the probability of detection for 1-min intervals varied by min, visit and location and ranged between 0.05 and 0.92 for both 6- and 10-min sampling periods (Figs. 4, 5). Mean detection probability ($\bar{p}$) per 1-min interval for both 10 and 6 min was lowest on 7 Jan., 2018 (0.29; 95% CI 0.24–0.34 for 10-min analysis and CI 0.22–0.37 for 6-min analysis) and highest on 1 Feb. 2017 (0.54; CI 0.46–0.61 and CI 0.43–0.64 for 10-min and 6-min, respectively). For 10 min of video, mean *p* per min was lowest at Wooten’s Pond (0.28; CI 0.19–0.41) and highest in canal 7 (0.50; CI 0.40–0.56). The correlation of detection by visit for 10 min ranged from 0.11 to 0.25. For 6 min of video, mean *p* per min was lowest at Big Cypress 1 (0.26; CI 0.18–0.35, based only on 2019) and highest in canal 7 (0.48; CI 0.40–0.55). The estimates of correlation of detection by visit for 6 min ranged from 0.12 to 0.28. Mean *p* for each min in the mitigation pools was lowest in 2019, the year in which significantly more manatees were using it.

#### Detection probability for 6 and 10 min of video

**Figure 6:**
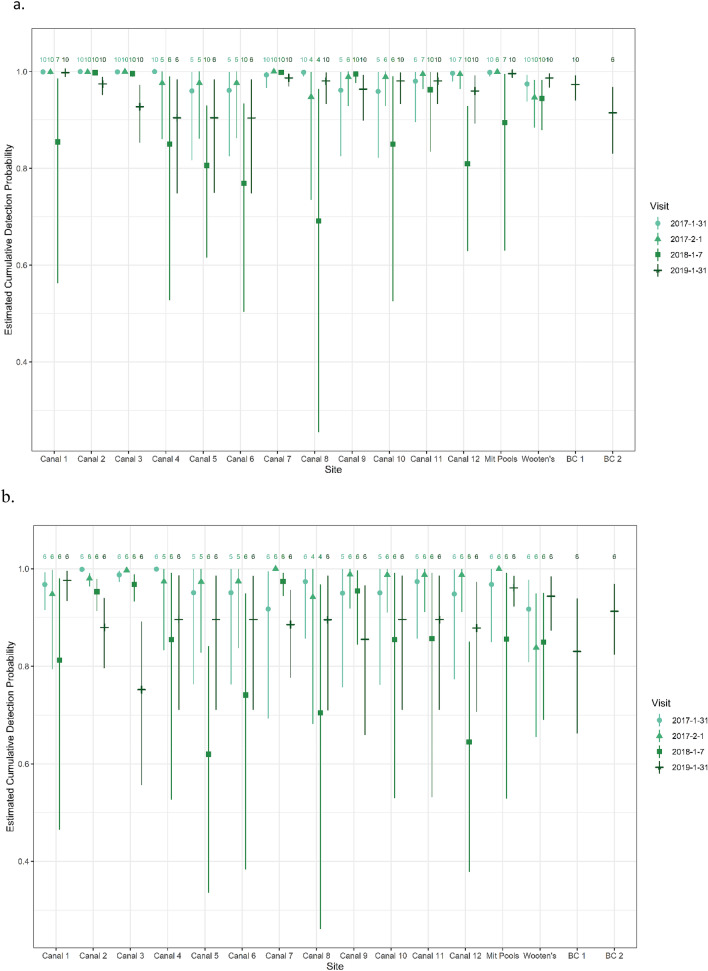
Estimates of the cumulative probability of detecting a manatee using (**a**) 6 min and (**b**) 10 min of video, by site and visit. Symbols indicate means (point estimates) and lines 95% credible intervals. The numbers at the top of the graphs indicate the total number of minutes in each survey. Site “Mit Pools” are the mitigation pools and “BC” indicates Big Cypress.

The cumulative detection probability at the site level using the 10-min sampling period was high, ranging from a low of 0.69 (CI 0.25–0.96) for canal 8 on 7 Jan. 2018 to a high of 1.00 for several locations on several dates (17 dates/locations, Fig. 6a). Detection using 6 min of video was also high, yet slightly lower than for 10 min (Fig. 6b).and ranged from a low of 0.62 (CI 0.3–0.84) for canal 5 on 7 Jan. 2018 to a high of 1.00 for 5 dates/locations. Generally, the detection was lowest in 2018, the coldest year (Figs. 2, 6).

### Abundance estimates

**Figure 7:**
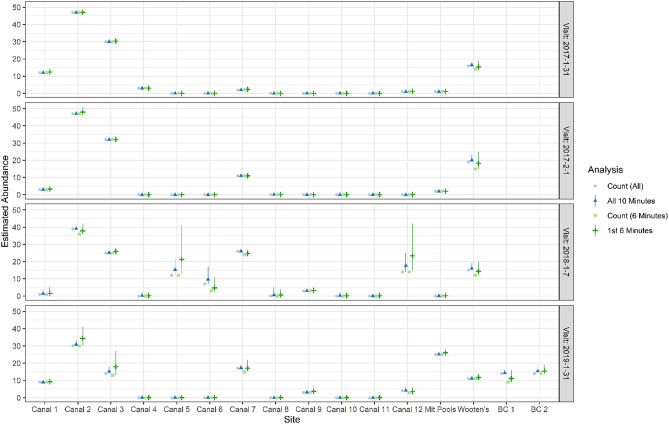
Estimates of abundance (triangles and crosses) and maximum counts (circles and squares) by site and visit for 6 and 10 min of video. Symbols indicate means (point estimates) and lines 95% credible intervals. Site “Mit Pools” are the mitigation pools and “BC” indicates Big Cypress.

**Figure 8:**
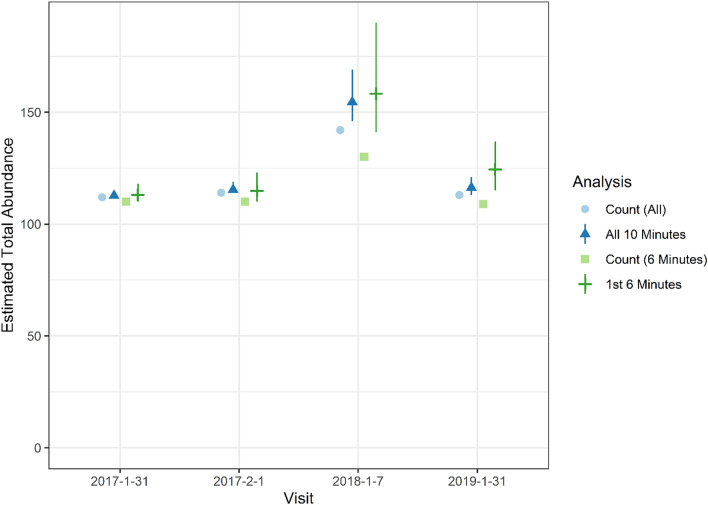
Total count (circles and squares) and abundance estimates (triangles and crosses) by visit, excluding Big Cypress. Symbols indicate means (point estimates) and lines 95% credible intervals.

Abundance for each site varied by survey (Fig. 7). The difference in detection between 10 and 6 min resulted in slight differences in estimates of *N* (abundance) between the two but greater uncertainty for 6 min (Fig. 7). The maximum difference in estimates between 6 and 10 min of survey time was 8 manatees (for 6 min, 31 Jan. 2019; Fig. 7), and the mean difference in estimates for all surveys was about 3 manatees. Due to high cumulative detection probabilities, the site-specific abundance estimates were seldom far above the raw counts, especially for the 2017 surveys and the full 10 min (Fig. 7). The mean difference between *N* and the counts was 1.0 for 6 min and 0.3 for 10 min; the maximum differences were 9 and 4, respectively. The estimate of the total abundance was highest in 2018 (6 min M = 158, CI 141–190 and 10 min M = 154, CI 146–169; Fig. 8), the coldest year (Fig. 2). Abundance was similar among the other years and time periods and ranged from *M* = 113–124 (Fig. 8). The abundance estimate correction by detection probability was more apparent for *M* than for *N*, and the maximum difference between *M* and total counts was 28 manatees for 6 min and 13 for 10 min (both in 2018). Canals 2, 3, 7 and Wooton’s Pond were used consistently by manatees (Fig. 7). The highest use was in canal 2 (*N* = 30–47); several canals were not used at all (#8 and #11) and others only occasionally (#4, #5, #6, #7, #10, mitigation pools; Fig. 1a,b). The mitigation pools were used by ≤ 2 manatees except during the last survey in 2019 when abundance was estimated to be 25 manatees (CI 25–26).

## Discussion

The thermal basins surveyed in this study help hundreds of manatees avoid cold stress syndrome in winter; few other passive thermal basins are used by manatees to this extent, and the nearest industrial warm-water site is more than 100 km away. The construction of the manatee mitigation feature at POI was the first attempt to create a permanent wintertime refuge for manatees. Therefore, it is important to accurately document the manatee population’s adjustments to the major changes in the warm-water habitat in this region. Furthermore, because anticipated reduction in the flow of fresh water to the POI basin and loss of the thermal halocline due to the PSRP raised concern about the potential for increased winter manatee mortality, getting precise estimates of the number of manatees using that basin and others nearby was important to those working to mitigate any negative impacts from the PSRP on this protected species. Using a UAS, we obtained high-quality videos that were used to identify manatees and create a capture history to help us model their abundance and distribution in the POI basin, the mitigation pools and other nearby refuge sites. Although we used only one observer to review the video, a second observer could be used for validation.

The UAS also reduced risk to aerial observers and reduced the cost of monitoring manatees at this location by at least one third. For data collection alone, our costs went from about $3000 per survey for the fixed-wing platform and observers, to about $2000 for the UAS pilot and video post-processing. Cost saving may be even larger in places where aircraft and pilots are hard to come by and more expensive to procure or if you operate your own UAS. Importantly, we developed and applied an innovative closed mark-recapture method using a Bayesian hierarchical framework to estimate abundance of manatees at each warm-water site with great precision while accounting for imperfect detection and avoiding errors of double counting and misclassification associated with it, something that had not been possible up to this point.

POI is primarily a thermal refuge where manatees rest in waters warmer than ambient to avoid cold stress. Because manatee’s resting behavior (time between surfacing interval or time spent on the surface or bottom) changes with environmental conditions such as air and water temperatures, wind, or physical characteristics of the canal such as depth, detection can change significantly between surveys or between locations within a single survey. This is especially true at POI where each canal varies in thermal quality and depth, and manatee use of each canal changes over time. Correlated surfacing behavior can also produce large differences in detection probability, even within a site and survey^25^. An individual manatee’s age or reproductive state also can influence its detectability; for example, younger animals or cows with calves may surface more frequently and thus be more available to be detected. Manatees can and do bottom rest for longer than 15 min, although this was not the case at POI, where surfacing intervals were observed to be much shorter. Our video indicated that manatees were available every couple of minutes at this location (much shorter than 6 min in most cases) and there was very little difference in estimates between 6 and 10 min. Regardless, by estimating the probability of availability our model accounts for imperfect detection. By reducing the sampling time to 6 min from 10 min we could reduce the amount of data collected, thereby increasing the uncertainty of those estimates, but they remained unbiased. However, our varied estimates of detection (Fig. 6) for each site does show that it is important to measure and account for site related differences when estimating abundance at manatee aggregations sites; this is likely the case for other species too.

Estimates of manatees within each site helped us identify which sites were most consistently used by manatees and by how many. Going forward this information will be integral for assessing changes in use at each of those sites as the halocline is eliminated and ambient water temperatures begin to drop below 18 °C in the boat basin during winter. Our results also indicated that after a few winters (a break-in period), manatees started to use the mitigation pools during cold periods—an important measure of success for this project. However, more monitoring is needed to determine the extent of their use in the future as the traditionally used warm-water refuge in the POI basin is eliminated.

Loss of warm-water habitat is one of the greatest threats to the Florida manatee population^33^. A goal of wildlife managers tasked with protecting manatees is to have reliable networks of warmwater habitat^7,9,34,35^. Methods for conducting surveys of aggregations have been inadequate for precisely estimating the numbers of manatees using these areas, which has made it difficult or impossible to assess changes in use of these sites over time. This is likely a problem for estimating abundance of other wildlife species as well, e.g., other sirenian species or aquatic animals such as hippopotamus or diving waterfowl. Using UAS-collected data to model abundance and distribution has broad applicability for surveys of other aggregated wildlife species in general^38–45^.

Our work shows that using a UAS platform to monitor aggregation sites can help provide reliable abundance estimates at locations that previously could be assessed only through less reliable techniques. The ability to effectively monitor an area is severely hampered where water clarity is poor, the number of aggregated animals is high and the fixed-wing flight path is difficult or dangerous to navigate. In addition, combining large-scale abundance surveys^26,36^ with UAS surveys of small aggregation sites could be used to estimate total population abundance over a wider area while also reliably determining the proportion of animals using the aggregation sites. In the case of Florida manatees, monitoring the use of warmwater has been an important goal of the Warmwater Habitat Action Plan^37^ aimed at addressing the loss of warmwater refugia, one of the most significant threats to the continued existence of the subspecies.

Although our approach can reduce several sources of errors, such as imperfect detection and non-independence of detection, there are some probable limitations. We assumed our surveyed areas were closed, whereas there may have been some movement into or out of canals during surveys that would change our estimates of detection. Our estimation of detection may be affected by factors such as heterogeneity of detection (e.g., animals with different diving patterns^1,24^) or extreme movement of individuals because the spatial location of an individual manatee is key to identifying it. Even though using UAS technology may help reduce misclassification errors (e.g., same animal counted twice; conversely, different animals could be misclassified as just one), such errors remain possible.

The uses of UAS in wildlife biology are varied but the ability to estimate population size is often fundamental for conservation biology. Many studies have incorporated the use of UAS to help observe behavior or to obtain high-quality video or photographs of target species. Fewer studies have used the platform to obtain data for modeling abundance and distribution for management purposes^11,12,15,26,36^. In our case, manatees can be elusive and estimating aggregations from a circling fix-winged aircraft can be difficult. It is especially difficult to count or photograph manatees for abundance estimation during observations taken over short time periods while an aircraft is tightly circling a small canal or water body (at an altitude of about 229 m or more). Prior to this study, obtaining accurate estimates of large manatee aggregations was limited by the difficulty in estimating detection of unmarked animals, for example, because of the amount of time it takes to survey these locations in a fixed wing aircraft and movement of animals between passes^18^. To get precise estimates, it was important for us to account for those individuals that might easily be missed by observers during surveys. In fact, previous challenges in estimating abundance of manatee aggregations was the reason the Florida Fish and Wildlife Conservation Commission (FWC) conducted its statewide abundance surveys in months when there were not many animals present in the warmwater refugia^26,36^. Our new methodology may help to obviate some constraints and future work will aim to assess applicability in very highly aggregated sites, which could be a useful complement to the approach used for the FWC statewide manatee abundance surveys. Advancements in the methods could lead to improving estimates of manatee warmwater carrying capacity^15,33^.

Although identifying individual manatees from the video was not difficult, manatees did move about. An important assumption of our model is that individuals can be accurately identified during each 1-min interval. Movement of animals over the 10-min time-period could have increased the possibility of making an identification error and increased the possibility of a closure assumption violation. Reviewing the video showed that the longer the period of observation, the more likely manatees were to move and the greater the distance they would travel. Using a 6-min period instead of 10-min period likely decreased the possibility of making an identification error but it gave up little in terms of precision in the estimates. Having a shorter time interval would also help conserve the UAS’s battery life (which is usually < 30 min) and help shorten time in the field. Days are shorter in the winter, and the best quality videos were obtained in the morning and mid-day when the angle of the sun was the highest and there was less interference from glare and sun glint. These considerations could be important for other studies that consider applying our methodologies to other species (e.g., alligator surveys).

As regulations concerning UAS change and technology advances, UAS will become an increasingly important tool for monitoring wildlife. The usefulness of the tool to assess abundance and distribution will be determined by the ability of scientists to develop reliable statistical models that account for bias and uncertainty. We have shown in this work how technology and innovative statistical modeling can be integrated to assess distribution and estimate abundance of a threatened species to help inform species management. Although we applied our methods to manatees, this approach can be applied to other systems and species that aggregate in large numbers (with minimal movement) such as crocodiles, hippopotamus, marine mammals, waterfowl and others. The method presented here can reduce costs of surveys, increase safety of observers and allow populations previously too difficult to monitor to be estimated in a rigorous way.

## Data Availability

The datasets generated during and/or analyzed during the current study are available from the corresponding author.
